# Limb Salvage and Survival in Chronic Limb-Threatening Ischemia: The Need for a Fast-Track Team-Based Approach

**DOI:** 10.3390/jcm12186081

**Published:** 2023-09-20

**Authors:** Giorgio Ventoruzzo, Giulia Mazzitelli, Umberto Ruzzi, Francesco Liistro, Alessia Scatena, Eugenio Martelli

**Affiliations:** 1 Vascular and Endovascular Surgery Unit, San Donato Hospital Arezzo, Local Health Authorities South East Tuscany, 52100 Arezzo, Italy; giulia.mazzitelli@uslsudest.toscana.it (G.M.); umberto.ruzzi@uslsudest.toscana.it (U.R.); 2 Interventional Cardiology Unit, San Donato Hospital Arezzo, Local Health Authorities South East Tuscany, 52100 Arezzo, Italy; francesco.liistro@uslsudest.toscana.it; 3 Diabetology Unit, San Donato Hospital Arezzo, Local Health Authorities South East Tuscany, 52100 Arezzo, Italy; alessia.scatena@uslsudest.toscana.it; 4 Department of General and Specialist Surgery, Faculty of Pharmacy and Medicine, Sapienza University of Rome, 155 Viale del Policlinico, 00161 Rome, Italy; eugenio.martelli@uniroma1.it; 5 Medicine and Surgery School of Medicine, Saint Camillus International University of Health Sciences, 8 Via di Sant’Alessandro, 00131 Rome, Italy; 6 Division of Vascular Surgery, Department of Cardiovascular Sciences, S. Anna and S. Sebastiano Hospital, Via F. Palasciano, 81100 Caserta, Italy

**Keywords:** peripheral arterial disease, critical limb ischemia, chronic limb-threatening ischemia, survival, mortality, limb salvage

## Abstract

Chronic limb-threatening ischemia (CLTI) represents the end-stage form of peripheral arterial disease (PAD) and is associated with a very poor prognosis and high risk of limb loss and mortality. It can be considered very similar to a terminal cancer disease, reflecting a large impact on quality of life and healthcare costs. The aim of this study is to offer an overview of the relationship between CLTI, limb salvage, and mortality, with a focus on the need of a fast-track team-based management that is a driver to achieve better survival results. This review can be useful to improve management of this growing impact disease, and to promote the standardisation of care and communication between specialist and non-specialist healthcare professionals.

## 1. Introduction

Peripheral arterial disease (PAD) is a global pandemic of growing proportions and increasing healthcare costs around the globe [1]. The Global Burden of Disease study reported that 202 million adults worldwide have PAD, a higher prevalence than ischemic heart disease (154 million), heart failure (64 million), Alzheimer’s disease/dementia (44 million), and cancer (43 million) [2]. Alarmingly, the prevalence of PAD will probably grow due to population aging and the growing prevalence of risk factors, in particular diabetes mellitus (DM). Between 2017 and 2045, the prevalence of DM is expected to rise from 451 to 693 million people worldwide (in 2040, 1 in 10 adults will have diabetes), and it is well known that DM increases the risk and severity of PAD [3]. 

According to the 2019 Global Vascular Guidelines (GVG) from the European Society for Vascular Surgery, advanced PAD is described as chronic limb-threatening ischemia (CLTI) that represents the end-stage form of the disease [4]. This new definition replaced the previous concept of critical limb ischemia (CLI) requiring an objectively documented atherosclerotic PAD in association with ischemic rest pain >2 weeks duration or tissue loss for diagnosis. The GVG recommend using objective hemodynamic tests, such as the ankle-brachial index (ABI) < 0.4, absolute ankle pressure (AP) < 50 mmHg, absolute toe pressure (TP) < 30 mmHg, transcutaneous pressure of oxygen (TcPO2) < 30 mmHg, and flat or minimal pulsatile volume recording (PVR) waveforms to determine the presence and to quantify severity of ischemia in all patients with suspected CLTI (Recommendation 1.1). In addition, the GVG stress the use of a threatened limb classification based on the presence and degree of tissue loss, ischemia, and infection (e.g., WIfI classification) that grades wound extent, degree of ischemia, and severity of infection to guide clinical management (Recommendation 1.2). CLTI definition, being accompanied by objective evidence of significant PAD (e.g., WIfI ischemia grade > 1), excludes purely neuropathic, traumatic, or venous ulcers lacking any ischemic component.

CLTI affects up to 10% of patients with PAD and is associated with significant mortality, pain, amputation rate, and impaired quality of life. Up to 50% of all patients with CLTI are diagnosed with DM, which is associated with lower revascularisation success rates, decreased wound healing, and higher amputation and mortality rates compared with those without diabetes [5].

CLTI generally results from involvement of at least two arterial segments (aorto-iliac, femoro-popliteal, tibio-pedal) or severe tibio-pedal disease alone. The latter is particularly involved in patients with DM, end-stage renal disease (ESRD), or very elderly. CLTI is a strong indication to endovascular, surgical, or hybrid revascularisation, in order to prevent major (above the ankle) amputation with the aim of preserving foot plantar support despite the need for minor (below the ankle) amputations, thus, obtaining limb salvage (LS).

## 2. CTLI Mortality and Amputation Rate

General and limb prognosis of these frail patients is adverse: they are at continuous risk of a major cardiovascular event, sudden death, and major amputation. When an individual first receives a diagnosis of CLTI, mortality risk is around 20–25% over 1 year, and around 60% over 5 years [6,7]. Reported 5-year all-cause and cardiovascular mortality rates were twice as high (57% and 29%, respectively) compared with patients with intermittent claudication (IC) (31% and 15%, respectively), according to a Dutch national registry study [8].

CLTI can be considered very similar to a terminal cancer disease. Few diseases connote a higher mortality rate. Data collected from American Cancer Statistics Center show that among 22 different types of malignancy, only 6 have a 5-year mortality rate higher than that of CLTI. Yet CLTI is even more deadly than this statistic suggests. Many cancers with high mortality rates are relatively rare, so the overall mortality burden to the population is modest; conversely, the mortality burden associated with some of the most common cancers is blunted due to relatively low mortality rates. Consequently, several deadly cancers, such as melanoma or ovarian cancer, are actually less common and less deadly than CLTI. Because CLTI is both common and deadly, more incident cases die during the 5 years after a CLTI diagnosis than with any type of cancer, except for lung cancer [9].

If left untreated, the overall risk of limb loss in CLTI is estimated at approximately 20–25% at 1 year, reflecting a large impact on quality of life and healthcare costs. More than half of people with a major amputation will be dead in 5 years [10,11].

A meta-analysis of 13 studies with 1527 patients on the natural history of untreated CLTI reported that at a median follow-up of 12 months, both the mortality and the amputation rates were 22%, although there was a marked heterogeneity between the studies [7].

In a study of 574 patients with CLTI who did not undergo revascularisation after 2 years, 31.6% had died, primarily of cardiovascular disease, and 23% required major amputation [12].

A recent study [13] investigated the long-term survival and amputation-free survival at 5 years in a cohort of 150 patients with non-revascularisable or so called “no option” CLTI. Amputation-free survival was 43% five years after inclusion. This outcome was driven by an equal rate of all-cause mortality (35%) and amputation (33%). Amputation occurred predominantly in the first year. Furthermore, 33% of those with amputation subsequently died within the investigated period, with a median interval of 291 days. Meloni et al. reported a 30% amputation rate and 50% mortality rate for no option CLTI diabetic patients at 1 year follow-up in a retrospective cohort study [14].

## 3. Limb Salvage and Mortality

Major amputation is an established risk factor for death. Perioperative mortality rate after below the knee amputation (BKA) is around 5–10% and rises to 15–20% after above the knee amputation (AKA). Five-year mortality rates of up to 85% have been reported in elderly CLI amputees, and seven-year rates after below and above the knee amputations in a veteran cohort published in 2003 were 72% and 80%, respectively [15,16].

Other studies showed a 3-year death rate of 33.3% after BKA, and 71.4% after AKA. At 5 years, these rates increased to 63.3% for BKA and 85.7% for AKA [17].

Despite the guidelines generally recommend to revascularise CLTI patients, the underlying evidence for such a recommendation is limited. However, if we consider the group of patients that undergo some kind of revascularisation in order to prevent major amputation, outcomes are more favourable [18].

A German study on a retrospective real-world cohort [19] comparing the outcomes of CLTI patients with and without revascularisation in a period between 2009 and 2011 showed that revascularisation is associated with significantly better short- and long-term outcomes in term of limb amputation (40.4% vs. 46.5%, respectively) and overall mortality (42.6% vs. 48.2%, respectively).

The Italian CLIMATE registry on 2399 patients treated for CLTI [20] documented an overall mortality of 3.1% at 30 days, and 13.5% at 1 year. Mortality did not statistically differ between genders even if females, who have less comorbidities but are significantly older (over-75), died more than males. Age seems to be a key determinant factor in the outcome of patients treated for CLTI. Age > 75 years, coronary artery disease (CAD), cerebrovascular disease (CVD), and major amputation at the first operation are independent negative prognostic factors for survival at short- and mid-term, as well as haemodialysis treatment and tissue loss for 1-year survival. These findings support the effort to attempt revascularisation in patients with CLTI, avoiding primary major amputation if possible. Approaches to this fragile population should, therefore, be directed towards aggressive risk factor control by using the best medical therapy in the long term, and strategies to decrease the amputation risk by means of timely evidence-based revascularisation in the short term, as pointed out from the GVG.

## 4. Medical Management Improving Survival in CLTI and Limb Salvage

CLTI is a terminal manifestation of systemic atherosclerosis; therefore, it is often accompanied by clinically significant CAD and CVD, resulting in exceedingly high mortality from stroke and myocardial infarction [21,22,23,24]. The goal of treating patients with CLTI is not only to save a still functional limb, but to reduce major adverse cardiac events (MACE) through aggressive risk factor modification and the best medical therapy. Whereas certain risk factors cannot be modified (such as age and sex), others can (DM, hyperlipidemia, hypertension, diabetes, smoking, and sedentary lifestyle). In the absence of aggressive identification and treatment of risk factors and associated comorbidities, the prognosis of CLTI is usually poor [25,26]. Sub-optimal medical therapy for comorbid conditions has been associated with up to 26% all-cause mortality rates within the first year of CLI diagnosis [4]. Therefore, nowadays, risk factors’ aggressive treatment is considered a cornerstone in CLTI management [27,28,29,30,31,32,33].

The GVG strongly recommend the best medical therapy, including the use of moderate- or high-intensity statin, antihypertensive, glycaemic control, and antiplatelet agents, to reduce all-cause and cardiovascular mortality in patients with CLTI, as well as counselling on smoking cessation, healthy diet and weight loss, regular physical exercise, and preventive foot care [4].

Novel oral anticoagulants (NOACs) are assuming an increasing role in reducing MACE and major adverse limb events (MALE) in PAD patients. According to the COMPASS study, a low dose of rivaroxaban (2.5 mg twice a day) plus 100 mg ASA determine a 28% reduction in MACE, a 46% reduction in MALE, and a 31% reduction in the composite endpoint occurrence rates compared to ASA, with no excess of fatal or critical bleedings [34]. The VOYAGER PAD trial demonstrated that dual therapy with low-dose rivaroxaban and aspirin significantly also reduces MACE and MALE occurrence in patients with symptomatic PAD undergoing revascularisation vs. aspirin alone (HR 0.85, 95% CI 0.76–0.96) [35].

## 5. The Need for Fast-Track Team-Based Management for Optimal CLTI Care

CLTI patients represent an extremely high-risk subset and deserve a proven clinical pathway. Limiting limb tissue loss in CLTI patients is of paramount importance in preventing the major amputations that are one of the major drivers to mortality in these patients; therefore, CLTI is a strong indication to revascularisation (endovascular, surgical, or hybrid) with two main targets, as follows:

(1) Clinical: prevent major amputation (above the ankle) and obtain LS, keeping plantar standing despite the need for minor (below the ankle) amputations.
(2) Technical: obtain direct flow on at least one tibial artery.

As stressed from the GVG [4], treatment should be achieved within 2 weeks from diagnosis. Despite the availability of different treatments and specific guidelines, patients with CLTI are often undertreated. Long-standing concern exists regarding late presentation and delayed management contributing to increased amputation rates. Multiple healthcare specialists are involved in the management of CLTI, yet a lack of public awareness and the frequent failure to make an early diagnosis continue to be major obstacles to effective treatment. Time delays in CLTI identification, referral, and management have a direct and detrimental impact on the outcome for the patient [36]. Societal guidelines recommend that all individuals diagnosed with CLTI undergo an imaging study to assess the viability of endovascular or surgical revascularisation, but variability in practice patterns is high, contributing to a broad disparity in the use of treatments and clinical outcomes. For example, a study from the United States suggested that many patients do not even receive angiography in the year before major limb amputation [37]. In the last two decades, improved awareness of the need for limb preservation has given rise to the idea of integrated amputation prevention programs in which specialized multidisciplinary teams cooperate in the medical/surgical management of these patients [38,39]. In the new GVG, the importance of multidisciplinary teams (MDT) and centres of excellence for amputation prevention are stressed as a key health system initiative [4]. Evaluation of peripheral vasculature and prompt revascularisation are, therefore, key components in managing CLTI.

In our institution in Arezzo, since the first decade of this century, we have strived to achieve excellence in the management of diabetic feet and CLTI by developing a fast-track team-based approach [40]. This was assured by the presence of a high-volume diabetic foot clinic with dedicated personnel and surgical competencies, an aggressive endovascular interventionalist attitude, and a daily collaborative interaction between different specialists: a diabetologist with special expertise in diabetic foot, a vascular specialist with special expertise in peripheral procedures (with either a vascular surgeon or an interventional cardiologist both in charge for endovascular revascularisation, and a vascular surgeon responsible for the open surgical approach), infectious disease specialists, and orthopaedic surgeons. Depending on the single-center organization, an angiologist and interventional radiologist can also be part of the multidisciplinary team. A podologist and two specially trained nurses are also essential members of the team. In addition to this, the foot clinic works closely with nurses of the community wound service that provide home wound care. Of note, the cardiovascular laboratory interventional team (including nurses and radiology technicians) and two cath labs are shared between the vascular surgeons and interventional cardiologists.

The implementation of a team approach has led to a systematic process for screening, evaluation, treatment, and follow-up of CLTI patient (with or without diabetes mellitus). These results have been certified by national evaluation, becoming a benchmark in Italy. The major amputation rate in the Arezzo area (350,000 inhabitants and more than 23,000 diabetic patients) in 2021, certified by Outcomes National Program (PNE-Programma Nazionale Esiti) is the lowest in Italy, corresponding to 0.01 × 1000 inhabitants with a national rate of 0.07 × 1000 [41]. Performance data from MeS Sant’Anna-Pisa, referring to 2022, reported a major amputations rate of 10.3 per million inhabitants with a Tuscan regional rate of 16.9 per million inhabitants [42].

The core strategy leading to these results has been the implementation of a fast-track approach centring on the proactive role of the foot specialist, and on the direct involvement in the diagnostic phase of the vascular specialist (either an interventional cardiologist or a vascular surgeon) that will take charge of the patient for the eventual endovascular treatment.

Patient can be referred to a foot clinic (mainly diabetic patients) or directly to a vascular surgeon (mainly non-diabetic patients) that will ask for a foot clinical evaluation in case of ulcerations/tissue loss.

**Figure 1:**
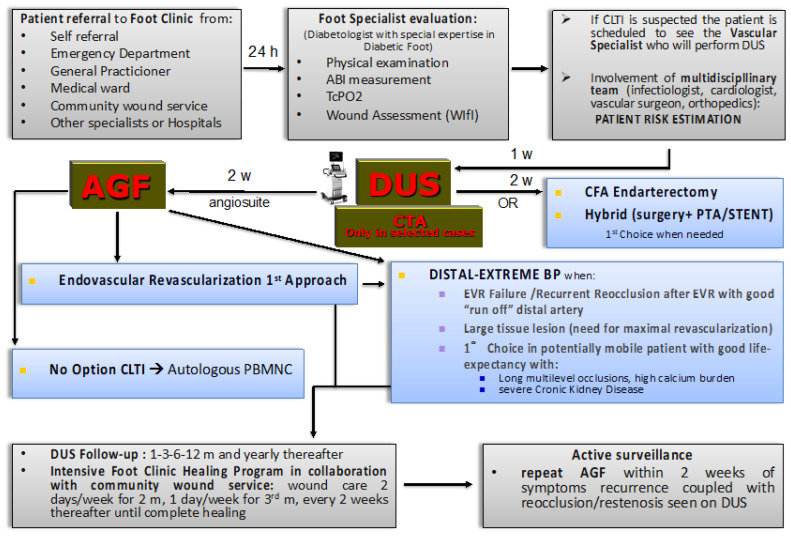
Algorithm of the Arezzo fast-track team-based management of CLTI patients with tissue loss. ABI, ankle-brachial index; TcPO2, transcutaneous oxygen pressure; WIfi, wound, ischemia, foot infection classification; CLTI, chronic limb-threatening ischemia; DUS, duplex ultrasound; AGF, angiography; CTA, computerized tomography angiography; CFA, common femoral artery; PTA, percutaneous transluminal angioplasty; BP, bypass; EVR, endovascular revascularisation; PBMNC, peripheral blood mononuclear cells.

Our treatment algorithm in the case of a CLTI patient with tissue loss referred to the “Foot Clinic” is summarized in Figure 1.

The foot clinic evaluation provides complete physical examination and non-invasive hemodynamic tests (including ABI, TcPo2, and continuous-wave Doppler) in order to collect objective parameters to define the degree of ischemia and establish the correct WIfI score. If CLTI is suspected by the foot specialist, the patient is scheduled to see the vascular specialist that will perform a rapid-access intervention-oriented duplex ultrasound (DUS) within maximum of 1 week. The vascular specialist is the same one who will eventually execute the possible angiography and eventual consensual endovascular revascularisation. DUS is a first-line imaging technique, and is the only preoperative imaging technique we use in the majority of cases. It allows us to detect associated vascular diseases (such as abdominal aortic aneurysm, or internal carotid artery stenosis), the location and extension of the lesions, the availability of autologous veins for eventual distal bypass, and helps in planning the surgical or endovascular intervention. Only a few cases need computed tomography angiography (CTA) to obtain essential information for planning. If a definitive diagnosis of CLTI is established, according to the recent GVG, LS is attempted within 2 weeks in all cases, after a careful planning of the interventional solutions, including the decision of whether the strategy can be completed in one or multiple sessions [4].

LS is associated with endovascular or open surgery revascularisation. The complex clinical scenario of CLTI patients, especially with diabetes or ERSD, requires integration between the vascular anatomy, the interventional possibilities, and the comorbidities of the patient in order to select the appropriate revascularisation strategy that has to be tailored on the single patient.

We follow an “endovascular-first” approach unless the vascular anatomy suggests a surgical or hybrid procedure (such as common femoral artery endarterectomy, with or without PTA/stenting of the iliac-femoral-popliteal-tibial arteries). Distal bypass to the foot is preferred as a first approach only for average-risk patients with advanced limb-threat and high complexity disease in the presence of a suitable great saphenous vein.

Notably, as underlined in the GVG, the first step that controls the treatment algorithm is the patient risk estimation [4]. Team evaluation of patient frailty, periprocedural risk, quality of life, and life expectancy leads to the LS candidacy decision. To define a strategy, it is paramount to consider the patient first, and not the lesion. That is the cornerstone of the patient-centred approach in a multidisciplinary team. Primary amputation or palliation should be offered to patients with limited life expectancy, poor functional status (e.g., non-ambulatory), or an unsalvageable limb after shared decision-making. Revascularisation as a palliative treatment should only be considered to improve inflow for a subsequent major amputation at the more distal level, or to relieve intractable pain.

“No option” CLTI patients, that are not eligible for revascularisation as a result of the inability to overcome vessel obstruction, no visible arterial circulation in the foot (“desert foot”), and/or for critical general conditions [14] can be candidates for autologous cell therapy with peripheral blood mononuclear cells (PBMNC), which has arisen as a possible strategy to relieve ischemic pain and promote ulcer healing before eventually taking into consideration a major amputation [43,44].

Target vessel patency is assessed by DUS before hospital discharge and at 1, 3, 6, and 12 months, and yearly thereafter.

Once discharged, all patients are followed by the foot clinic in order to promote the healing process and deambulation function recovery, two days per week for the first two months, once a week for the third month, and every two weeks until complete healing. Pre-intervention planned minor amputations are usually performed 2–4 weeks after revascularisation and include finger amputations and metatarsal amputation due to necrosis/infection of tissues and bones, with particular preservation of healthy and surrounding tissues.

The foot specialist plays a crucial role in dictating the indication to repeat revascularisation, alerting the vascular specialist regarding the reoccurrence of rest pain and negative outcome of the ulcer. The foot specialist also receives continuous feedback from nurses of the community wound service that provide home wound-care. No healing, onset of new lesions, or worsening of the wound (increase, necrotic margin, fibrin deposition, regression of granulation tissue) detected during wound medication sessions prompt DUS evaluation of the treated or contralateral limb. If re-occlusion or restenosis are detected, angiography and repeat revascularisation is scheduled in a fast-track fashion within 2 weeks.

A recent paper investigating mortality in paclitaxel eluting devices (PED)-treated patients compared to non-eluting devices (NED) gave us the chance to retrospectively revise our previous 10-year experience on limb salvage, highlighting the effect of a fast-track strategy on major amputation rate [45]. During the study period, 3450 procedures in the lower limb in 1521 patients were performed. Among these, 1294 (718 NED and 576 PED) met the inclusion/exclusion criteria and entered in the study. Seven hundred (54%) of these patients were enrolled in previous clinical trials [46,47,48,49,50]. Follow-up length was 58 ± 34 months. Relapse of CLTI occurred in 120 (29%) patients in the PED group and in 141 (32%) patients in the NED group (*p* = 0.3). Among these patients, 108/120 (90%) in PED and 132/141 (93.6%) in NED underwent repeat intervention of the target limb (*p* = 0.3). The major amputations rate at 7-year was very low (2.3% in NED, 1.6% in PED). This low major amputation rate is probably a consequence of the dedicated multidisciplinary clinical pathway for CLTI patients, the fast-track strategy for treatment, and eventual re-treatment, as well as the intensive foot-healing program assuring continuous monitoring of healing and patency.

## 6. The Impact of Paclitaxel-Eluting Devices (PED) on Mortality

The advent of drug-eluting technology with PED significantly reduced the restenosis rate and the need for target lesion redo endovascular treatment of CLTI patients [46,47,48,49,50,51].

A meta-analysis of randomized trials of PED for femoropopliteal interventions published in 2019 reported a safety issue related to a two-fold increase in 5-year mortality in patients treated with PED, compared to non-eluting devices (NED) [52]. The mechanism responsible for late mortality remained unknown. Reasonable doubts have been raised on methodical issues (such as a lack of information on the original patient data, cross-over, relevant loss of follow-up in the RCTs, incomplete RCT data reporting), which has caused a worldwide scientific debate. The FDA recommended the use of treatments other than paclitaxel-coated balloons and stents for most PAD patients. In response to this safety signal, several studies on large health-care databases and patient-level data of the single RCTs included in the meta-analysis were published, and PED were associated with a significant increased mortality risk in none of them [53,54,55,56].

An insight from the Voyager PAD RCT has clearly demonstrated no difference in long-term mortality according to the use of PED or NED, with an all-cause mortality of 12.9% in the NED group vs. 12.1% in the PED group at 42 months from randomisation [57].

In our center focused on limb salvage, we recently investigated mortality in PED-treated patients [45]. Results suggested a clear reduced mortality for PED compared to NED treatment in a real-world CLTI scenario at 2 years (12% vs. 18%, respectively) and 5 years (30% vs. 36%, respectively). This advantage tends to disappear at 7-year follow-up due to the reduced life expectancy of >75 years old patients.

## 7. Conclusions

The impact of CLTI on the affected patient is comparable to that of cancer, and the costs involved in the management of this widespread disease are enormous. Despite the availability of different treatments and specific guidelines, patients with CLTI are often undertreated. Limiting limb tissue loss is paramount in preventing major amputation, which is one of the major drivers to mortality in this subset of patients. A fast-track strategy, with less than 2-week interval between diagnosis and treatment, together with continued wound care, healing process evaluation, appropriate medical therapy and immediate evaluation in case of possible CLTI recurrence, are the keys to success.

The experience of single members of the team is also a cornerstone of management success. Notably, this field of action is a great challenge for vascular surgeons that need to acquire advanced DUS performing skills, high expertise in both endovascular and open surgery lower limb revascularisation, an aggressive but also versatile planning attitude in order to tailor interventional strategies for individual patients, and an open mind oriented with an eye on cost/effectiveness evaluations. Vascular surgeon societies should have an active role in promoting this educational goal.

## Data Availability

Not applicable.
